# Sodium-dependent glucose transporter 1 and glucose transporter 2 mediate intestinal transport of quercetrin in Caco-2 cells

**DOI:** 10.29219/fnr.v64.3745

**Published:** 2020-06-15

**Authors:** Suyun Li, Jin Liu, Zheng Li, Liqin Wang, Weina Gao, Zhenqing Zhang, Changjiang Guo

**Affiliations:** 1 Beijing Shijitan Hospital, Capital Medical University, Beijing, P R China; 2 Department of Nutrition, Tianjin Institute of Environmental and Operational Medicine, Tianjing, P R China; 3 Systems Engineering Research Institute, Beijing, PR China; 4 Beijing Institute of Pharmacology & Toxicology, Beijing, PR China; 5 Department of Epidemiology and Statistics, Hebei Medical University, Shijiazhuang, PR China

**Keywords:** absorption, Caco-2 cell monolayer, flavonoid, phloridzin, phloretin, transmembrane transport

## Abstract

**Background:**

The role of glucose transporters in the transport of flavonoids remains ambiguous.

**Objective:**

In this study, we examined whether quercitrin would be absorbed intactly in modeled Caco-2 cells, as well as determined the involvement of sodium-dependent glucose transporter 1 (SGLT1) and glucose transporter 2 (GLUT2) in its transmembrane transport.

**Design:**

The first experiment was conducted to examine the uptake of quercitrin into Caco-2 cells 24 h after they were seeded and the second experiment was conducted to determine the transport across the apical and basolateral membrane of Caco-2 cells after they were cultured for 21 days in a Millicell system. Quercitrin was administered at 3, 9, or 18 μg/mL; and the time points of sampling were 30, 60, 90, 120, and 150 min.

**Results:**

In the uptake experiment, the highest intracellular quercitrin concentration was observed in the cells treated with 18 μg/mL quercitrin at 60 min, with a bell-shaped kinetic curve. Quercetin, isorhamnetin, and tamarixetin were detected inside the cells, particularly when treated with a high dose. In the transport experiment, quercitrin was transported from the apical to basolateral side and vice versa; its concentrations depended on dose, time, and transport direction (*P* < 0.0001). Only trace amounts of isorhamnetin and tamarixetin were detected in the apical chamber when quercitrin was added to the basolateral chamber. Phloridzin and phloretin, potent inhibitors of SGLT1 and GLUT2, respectively, significantly diminished quercitrin transport from the apical to basolateral side; and phloretin had a greater inhibitory effect compared to phloridzin.

**Conclusion:**

Our results demonstrate that quercitrin is absorbed intactly and then effluxed out of Caco-2 cells through both apical and basolateral membranes probably via SGLT1 and GLUT2.

## Popular scientific summary

- Both SGLT1 and GLUT2 in Caco2 cells are involved in transport of quercitrin, a flavonoid glycoside.
- The data of this study implicate flavonoid glycosides possibly have a direct effect on health.
- Whether such an observation will be replicated in other intestinal cell lines warrants future studies.

Both SGLT1 and GLUT2 in Caco2 cells are involved in transport of quercitrin, a flavonoid glycoside. These data implicate that flavonoid glycosides possibly have a direct effect on health. However, whether such an observation will be replicated in other intestinal cell lines warrants future studies.

Flavonoids, including flavones, flavonols, flavanones, flavanonols, flavanols, anthocyanins, and isoflavones, are ubiquitous in plant foods, for example, fruits, vegetables, and nuts, and they constitute an integral part of human diet. The growing body of preclinical, clinical, and observational evidence supports the fact that their consumptions are inversely associated with risk of chronic diseases, including cardiometabolic diseases, certain cancers, type 2 diabetes, neurodegenerative diseases, and cognitive decline (1–3) These health benefits of flavonoids are exerted through a wide array of putative bioactions, such as antioxidation, anti-inflammation, glucoregulation, and anti-proliferation (4–6). It is worth noting that the bioefficacy of flavonoids in target tissues depends on bioavailability, as well as bioaccessibility in the gastrointestinal tract (7).

Flavonoids mostly exist in glycosylated forms (glycones), with conjugates attached to their hydroxyl moieties (8). It has been illustrated that glycosylated moiety(ies) attached to flavonoid aglycones affects their absorption, distribution, and metabolism (6). It remains unclear which form (glycone vs. agyclone) is absorbed in the gastrointestinal tract even though it has been commonly accepted that most flavonoid glycosides, except anthocyanins, are only absorbed after the removal of the glycosidic moiety(ies) by enzymes in the small intestine (e.g. β-glucosidase and lactase phlorizin hydrolase) or microbial enzymes in the large intestine (e.g. α-arabinofuranosidase and α-rhamnosidase) (9). However, a few studies had reported the detection of flavonoid glycosides in the circulation (10–12) even though the evidence of the presence of these flavonoid glycosides has been questioned (13) because due to their hydrophilic characteristics, they do not diffuse across the apical intestinal membrane (14).

The absorption of flavonoids in the intestine can be mediated by active transport, passive diffusion, or both (15). Sodium-dependent glucose transporter 1 (SGLT1) has been reported to play a role in the cellular entry of flavonoids (16, 17). This transporter is mainly expressed in the apical membrane of intestinal epithelial cells, using Na^+^/K^+^-ATPase. Glucose transporter 2 (GLUT2) is mainly expressed in the basolateral membrane and mediates the transport of glucose into the circulation (18). However, the role of GLUT2 in the transport of flavonoid glycosides remains ambiguous at best (19, 20). It is very likely that GLUT2 is involved in the transport of quercetin because Farrell et al. (21) observed that flavonoid glycosides attenuated GLUT2-mediated glucose transport in a Caco-2 cell model.

Quercetin, a flavonol, is commonly found as quercetin glycosides in fruits, vegetables, red wine, and tea (22). Quercitrin (thujin or quercetin 3-rhamnoside), a quercetin glycoside, is commonly found in plant foods used to treat inflammation, diarrhea, and infection (23–28). In our knowledge, the information on quercitrin absorption is scarce in the literature. In a previous study involving rats, Morand noted that as compared to quercetin, rutin, and quercetin 3-O-glucoside, no quercitrin-derived flavonoid or metabolites were detected in plasma (16). Thus, we examined in this study whether quercitrin would be absorbed in human Caco-2 cells. Caco-2 cells are commonly used in flavonoid absorption studies in the literature. Furthermore, we examined the role of SGLT1 and GLUT2 in the transport of quercitrin. The results of this study can be helpful for the interpretation of physiological data, particularly those generated in cultured non-intestinal cells treated with flavonoid glycones or plant extracts.

## Methods and materials

### Reagents and instruments

Quercetin, quercitrin, isorhamnetin, tamarixetin, L-glutamine, and trypsin were purchased from Sigma (USA). HPLC-grade methanol was obtained from Thermo Fisher (Waltham, MA, USA), Dulbecco’s Modified Eagle’s medium (DMEM) from Gibco (Waltham, MA, USA), and fetal bovine serum and nonessential amino acids from Hyclone (Logan, *UT,* USA). Ultrapure water was prepared by using a MilliPore ultrapure water purifier (St. Louis, MO, USA). All other chemicals used in the experiments were of analytical grade. The 12-well Millicell plates were purchased from Millipore. LC/MS (Agilent 1100 LC/MS DVL, DE) was obtained from Agilent (Santa Clara, CA, USA).

### Quercitrin preparation

Quercitrin, quercetin, isorhamnetin, and tamarixetin were prepared in DMSO at the stock concentration of 4 mg/mL and stored at −80°C until use. The quercitrin concentrations to be administered were prepared by diluting quercitrin stock solution with PBS. DMSO concentrations in all administered quercitrin doses were smaller than 4%, a concentration displaying an insignificant (<5% inhibition) impact on cell viability as measured by using the MTT assay. Quercitrin at concentrations up to 100 μg/mL did not significantly affect cell viability.

### Cell culture

Human colon adenocarcinoma-derived Caco-2 cells were obtained from the American Type Culture Collection (ATCC, Manassas, VA, USA). The cells were grown in 75 cm^2^ flasks with DMEM containing 10% fetal bovine serum, 1% NEAA, 1% L-glutamine, and penicillin-streptomycin at 37°C in a humidified atmosphere having 5% CO_2_. The medium was changed three times weekly and cells were passaged at 75%–85% confluence.

### Quercitrin uptake in Caco-2 cells

Caco-2 cells were seeded at a density of 1 × 10^6^ cells/mL in 12-well tissue culture plates for 24 h. After removal of the medium, the cells were treated with quercitrin in 10 mmol/L PBS (pH 7.4) at the concentration of 0 (vehicle solution), 3, 9, or 18 μg/mL for 0, 30, 60, 90, 120, or 150 min. Both the concentrations and incubation times were deemed physiologically probable in foods containing flavonoids. All treatment conditions were conducted in triplicate. After removal of the medium containing quercitrin, the cells were washed twice with PBS, harvested, and then mixed with 200 μL methanol. After centrifugation, 60 μL supernatant was diluted with 240 μL methanol for the quantification of quercitrin, quercetin, isorhamnetin, and tamarixetin using LC/MS. Cellular protein content was quantified using Lowry’s assay to standardize flavonol contents.

### Transmembrane transport of quercitrin in Caco-2 cells

The experiment was conducted according to the previously reported protocol (29). Caco-2 cells were first cultured in 12-well Millipore Millicell (transwell) plates for 21 days. Transepithelial electrical resistance (*TEER*) was measured using a Millicell-ERS device (Millipore, Burlington, MA, USA) to confirm the integrity of the monolayer. TEER values of all wells used in the experiment were above 500 Ω.cm^2^. After removal of the medium, the cells were acclimatized to PBS buffer (pH 7.2) for 15 min at 37°C. Quercitrin was then added to the apical chamber to assess the transmembrane transport of quercitrin from the apical (A side) to the basolateral (B side) side or to the basolateral chamber to assess the opposite transport. Six hundred microliters of PBS (pH 7.2) containing quercitrin at the concentration of 0 (vehicle solution), 3, 9, or 18 μg/mL was added to the apical chamber and 1200 μL PBS to the basolateral chamber. Subsequently, 50 μL PBS in the basolateral chamber of the same wells was serially aliquoted at 30, 60, 90, 120, and 150 min and replaced with an equal volume of PBS. After the addition of the internal standard (buspirone in methanol), followed by vortexing, the resulting mixture was archived for the quantification of flavonols. To test the transport of flavonols from the B side to the A side, quercitrin in 1200 μL PBS was added to the basolateral chamber, followed by the same medium collection schematics.

When the inhibitors of glucose transporters were employed to assess their impact on A-to-B transport of quercitrin, 300 μL of 0.2 mmol/L phloridzin or phloretin (the final concentration was 0.1 mmol/L) was added to the apical chamber 15 min prior to the administration of quercitrin (18 μg/mL) at the apical chamber (30). The vehicle solution was used as a blank control where it was applicable.

### Flavonol analysis using LC/MS

Flavonols in the collected samples were determined using an Agilent LC-MS system, equipped with an Agilent-C18 column (2.1 × 100 mm, 3.5 μm). The column temperature was set at 25 ± 2°C. The mobile phase A (MPA) comprised 0.1% formic acid in water and the mobile phase B (MPB) comprised methanol and acetonitrile (V:V = 1:1) containing 0.1% formic acid. The elution of flavonols was achieved using an isocratic mode with the mobile phase comprising 65% MPA and 35% MPB at the flow rate of 0.2 mL/min. The injection volume was 20 μL. Mass spectrometer conditions were set as follows: electrospray ionization (ESI) mode, nebulizer pressure at 30 psi, drying gas flow at 8.0 L/min, drying gas temperature at 35°C, capillary voltage at 3000 V, quadropole temperature at 99°C, gain at 1, and peak width at 0.10 min. To improve the sensitivity and selectivity, selected ion monitoring (SIM) mode was used to monitor the compounds of interest. Fragmentor value of the monitored compounds and their mass-to-charge ratios (*m*/*z*) were first optimized using the flow injection analysis (FIA) in a full-scan mode. The *m*/*z* value of quercetin and quercitrin was 303 and 449, respectively. Both isorhamnetin and tamarixetin had the same *m*/*z* value of 317. The *m*/*z* value of buspirone, used as the internal standard, was 386.2. The calibration curve for quercitrin, isorhamnetin, and tamarixetin showed a good linearity with *r* > 0.99 in the range of 1–1000 ng/mL and for quercetin in the range of 2–2000 ng/mL.

### Statistical analysis

All data are expressed as mean ± SD. For the data related to both quercitrin uptake and transmembrane transport experiments, statistical significance was assessed using variance analysis of repeated measurement and variance analysis of factorial design, followed by Fisher’s *least significant difference (*LSD) test with Bonferroni adjustment for multi-comparison. The dependent variables for the analysis of the quercitrin uptake data included time, dose, and their interaction, and for the transmembrane experiments included time, transport direction (A to B or B to A), dose, and their interactions. *P*-value < 0.05 was considered statistically significant. All tests were performed using SPSS21.0 (SPSS, Inc., Chicago, IL, USA).

## Results

### Intracellular concentration of uptaken quercitrin

**Fig. 1:**
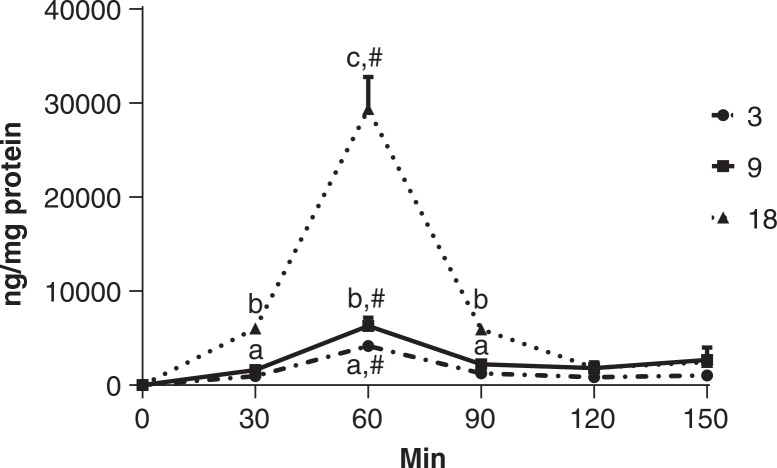
The uptake of quercitrin by Caco-2 cells. Quercitrin at the concentration of 3, 9, or 18 μg/mL is administered to the cells 24 h after being seeded. Vehicle containing no quercitrin was also administered but the data were depicted in the figure because quercitrin was not detected. Quercitrin is quantified using LC-MS in cell lysates at 30, 60, 90, 120, and 150 min post administration. Values are expressed as mean ± SD (*n* = 3). ^abc^Means at the same timepoints without sharing the same letter significantly differ (*P* < 0.05), tested by variance analysis of factorial design, followed by Fisher’s *least significant difference (*LSD) test with Bonferroni adjustment for multi-comparison. ^#^Mean within the same dose group is significantly different from others at different timepoints (*P* < 0.05).

In the uptake experiment in which Caco-2 cells were grown for 24 h prior to quercitrin administration, quercitrin was detected inside Caco-2 cells, and its intracellular concentrations were dose and time-dependent (*P* < 0.0001). As shown in Fig. 1, the highest quercitrin concentration was observed in the cells treated with 18 μg/mL quercitrin at 60 min; and the value of the high dose was 366.2% larger than the medium dose at the same time point (*P* < 0.05). The largest intracellular quercitrin concentrations of the two lower doses within their respective time course were also noted at 60 min. Only at 60 min time point, quercitrin concentrations were significantly different among three doses (*P* < 0.05), and at 30 and 90 min time points, the concentrations of two lower doses were not different from each other, but significantly different from the high dose (*P* < 0.05). At both 120 and 150 min time points, intracellular quercitrin concentrations were not different between three doses.

### Transmembrane transport of quercitrin

**Fig. 2:**
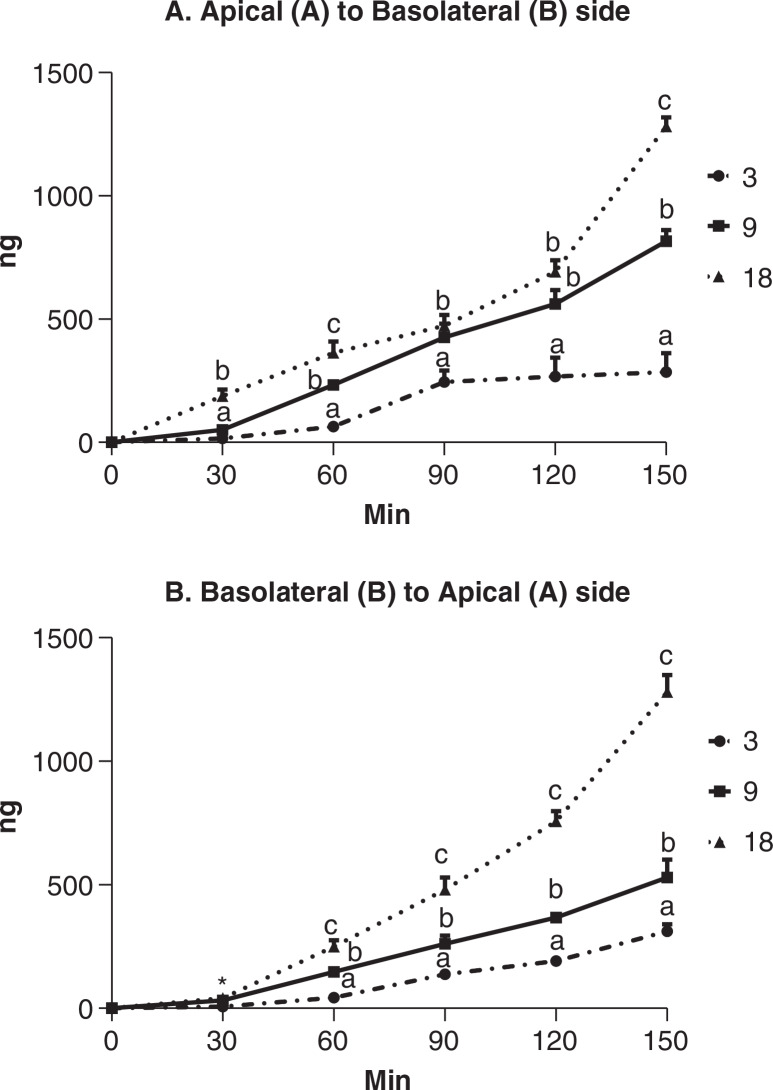
The transport of quercitrin by Caco-2 cells cultured in a Millicell (transwell) culturing system for 21 days, (A) quercitrin at the concentration of 3, 9 or 18 μg/mL is added to the apical chamber and quantified in the basolateral chamber and (B) quercitrin of the same concentrations is added to the basolateral chamber and quantified in the apical chamber. Vehicle containing no quercitrin was also administered but the data were shown in the figure because quercitrin was not detected. Quercitrin is quantified using LC-MS in the collected culture media at 30, 60, 90, 120, and 150 min post administration. Values are expressed as mean ± SD (*n* = 3). ^abc^Means at the same time points without sharing the same letter significantly differ (*P* < 0.05), tested by variance analysis of repeated measurement, followed by Fisher’s *least significant difference (*LSD) test with Bonferroni adjustment for multi-comparison. *At 30 min, the concentration of the high and middle doses are larger than the low dose and those of the high and middle are not different. The effect of transport direction, time, and dose factors on quercitrin transport is significant (*P* < 0.0001).

Quercitrin was detected in the chamber opposite to the administered chamber. Its concentrations were dependent on dose, time, and transport direction (*P*< 0.0001, 0.0001, and 0.001, respectively). Unlike the results of the uptake experiment and independent of the chamber of quercitrin administration, the ascending trend of quercitrin transport continued at the end of the experiment time of 150 min (Fig. 2A and B). From the B to A transport, quercitrin concentrations were different between three doses at all time points except the 30-min one (*P* < 0.05). From A to B transport, the difference between three doses at the test time points appeared not to be so distinctive. Out of five test time points, quercitrin concentrations at 30, 90, and 120 min were not different between doses of 9 and 18 μg/mL. The overall effect of the direction of quercitrin transmembrane transport was significant, but the main driver for the observed effect was attributed to the middle dose with quercitrin concentrations from A to B side at 30, 60, 90, 120, and 150 min at least tending to be greater than those from B to A sides (*P* = 0.081, 0.002, 0.011, 0.005, and 0.004, respectively).

### Quercitrin metabolites

**Fig. 3:**
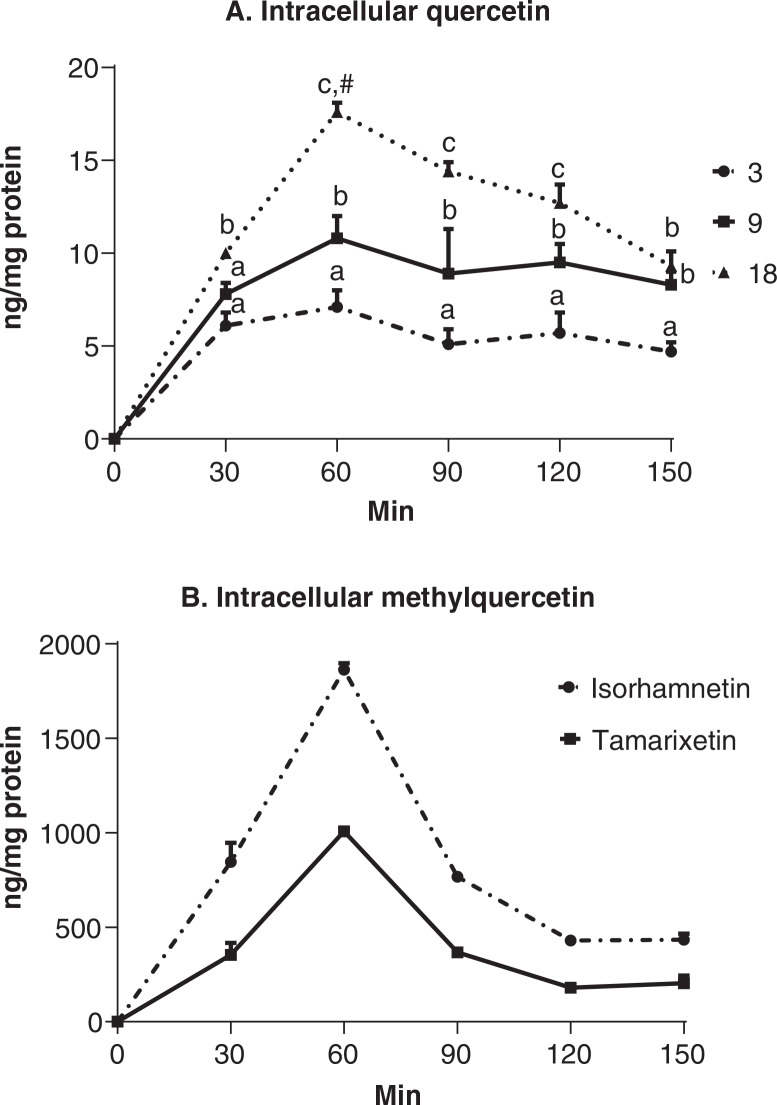
Intracellular quercetin, isorhamnetin, and tamarixetin in Caco-2 cells treated with quercitrin at the concentration of 3, 9, or 18 μg/mL. Vehicle containing no quercitrin was also administered but the data were shown in the figure because isorhamnetin and tamarixetin were not detected. The experiment is conducted in the cells that are cultured for 24 h after being seeded. Flavonoids are quantified using LC-MS in cell lysates at 30, 60, 90, 120, and 150 min post administration. Quercetin is detected in the cells treated using all three doses and isorhamnetin and tamarixetin are only detected at the high dose. Values are expressed as mean ± SD (*n* = 3).^abc^Means at the same time points without sharing the same letter significantly differ (*P* < 0.05), tested by variance analysis of factorial design, followed by Fisher’s *least significant difference (*LSD) test with Bonferroni adjustment for multi-comparison. ^#^Mean within the same dose group is significantly different from others at different time points (*P* < 0.05).

In the uptake experiment, quercetin, the deglycosylated counterpart of quercitrin, was detected inside Caco-2 cells from 30 min forward (Fig. 3A). Both time and dose factors had a significant effect on intracellular quercetin concentration (*P* < 0.0001). Quercetin reached the highest concentration at 60 min for all three doses but only the highest dose was significantly different from other time points in the intra-dose comparison. At 60, 90, and 120 min time points, quercetin concentrations were significantly different between three doses (*P* < 0.05). As compared to the other two doses, the highest dose displayed a clear increase and decrease pattern with the 60-min time point being the peak. Quercetin concentrations of both 3 and 9 μg/mL quercitrin doses remained comparable from 60 to 150 min. Isorhamnetin and tamarixetin, two methylation metabolites of quercetin, were only detected in the cells treated with 18 μg/mL quercitrin (Fig. 3B), but not in those treated with the lower doses. The intracellular amounts of these two metabolites were time-dependent, which peaked at 60 min, followed by a gradual decline along with the incubation times. The content of tamarixetin was significantly higher than that of isorhamnetin at all time points (*P* < 0.05). Their concentrations were also at least ~15-fold smaller than quercetin at the same dose at 60 min.

In the transmembrane transport experiments, trace amounts of isorhamnetin and tamarixetin (<1 ng/mL medium) were detected at 90 min in the apical chamber when quercitrin was added to the basolateral chamber. However, quercetin was not detected in the medium.

### The impact of phloridzin and phloretin on quercitrin transport

**Fig. 4:**
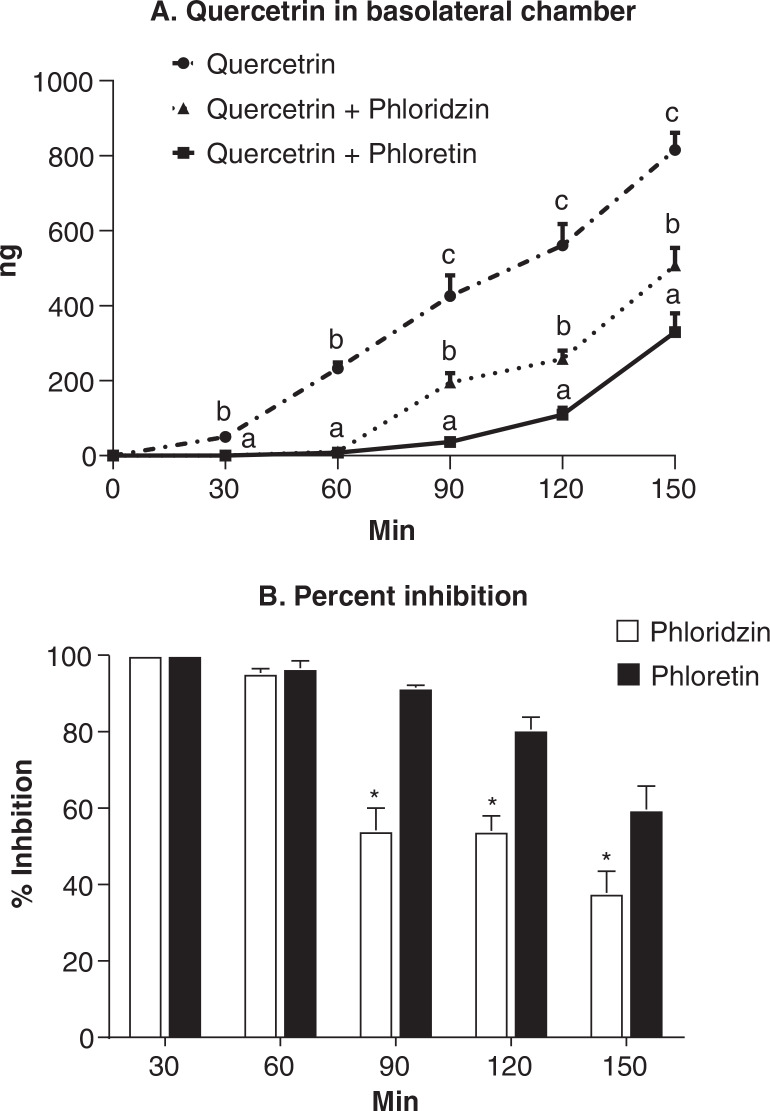
The apical to basolateral transport of quercitrin at 18 μg/mL by Caco-2 cells cultured in a Millicell (trans well) culturing system for 21 days and then pretreated with phloridzin and phloretin for 15 min, (A) time course of quercitrin transport and (B) percent inhibition of quercitrin transport. Quercitrin concentration is Phloridzin and phloretin is a non-specific, inhibitor of SGLT1 and GLUTT2, respectively. Vehicle containing no quercitrin was also administered but the data were shown in the figure because quercitrin was not detected. Quercitrin in the basolateral media is quantified using LC-MS at 30, 60, 90, 120, and 150 min post administration. The percent inhibition is calculated using the formula: (*Q* – *Q*+)/*Q* × 100%, where *Q* is the quercitrin concentration of the cells without the pretreatment of inhibitor and *Q*+ is quercitrin concentration of the cells with the pretreatment of inhibitor. Values are expressed as mean ± SD (*n* = 3). ^abc^Means at the same time points without sharing the same letter significantly differ (*P* < 0.05), tested by variance analysis of repeated measurement, followed by Fisher’s *least significant difference (*LSD) test with Bonferroni adjustment for multi-comparison. *Means at the same time points significantly differ (*P* < 0.05).

To demonstrate potential roles of SGLT1 and GLUT2 in the transport of quercitrin across the Caco-2 cell monolayer, we investigated the inhibitory effect of phloridzin, a potent inhibitor of SGLT1, as well as phloretin, a potent inhibitor of GLUT2, on the transmembrane transport of quercitrin. After their application in the apical chamber prior to quercitrin administration, both phloridzin and phloretin significantly lowered the transport of quercitrin (*P* < 0.0001) (Fig. 4A). The inhibitory potency between the two inhibitors appeared time-dependent. At 30- and 60-min time points, both inhibitors appeared to completely block the transport of quercitrin from the apical side to the basolateral side. The inhibitory effects of both inhibitors became weaker beyond 60 min of the incubation time, with phloretin being more potent than phloridzin (*P* < 0.05) (Fig. 4B).

## Discussion

Bioefficacy of flavonoids in plant foods in target tissues after oral consumption may be weaker than that reported in *in vitro* studies (31–33). This understanding began with the elucidation of their pharmacokinetic characteristics, including absorption, metabolism, disposition, and elimination, over the last 20 years (7). In addition, flavonoid glycosides are generally thought to be absorbed only after the removal of the glycosidic moiety(ies). However, we observe in this study that quercetrin uptake is seen in modeled Caco-2 cells which display enterocyte phenotype of the colon and small intestine. Our study also shows that the transport of quercetrin can be mediated by 2 glucose transporters, SGLT1 and GLUT1 (18).

It was speculated by Hollman et al. (34) in 1995 that quercetin glycosides might be actively absorbed in the human intestine via unspecified hexose transporters. A few years later, Gee et al. (35) reported that quercetin glucosides interacted with the sodium-dependent glucose transport for their entry into the enterocytes. Using the glucose transporter inhibitors, phloridzin and phloretin, which are potent, but non-specific inhibitors of SGLT1 and GLUT2, respectively (36, 37), Zou et al. (30) reported that both SGLT1 and GLUT2 contributed to the absorption of cyanidin-3-glucose. Furthermore, Walgren et al. (16) observed that both glucose and phloridzin inhibited quercetin-4-glucoside transport across the apical membrane of Caco-2 cells via SGLT1. Consistent with these results, our results confirm the transport of quercitrin in modeled Caco-2 cells and also support the involvement of both SGLT1 and GLUT2 in this process. Interestingly, all these data, including the present study, are conflicting with the results of Crespy et al.’s (38) study, in which the authors reported no contribution of SGLT1 to the intestinal transport of isoquercitrin (quercetin 3-O-glucoside) in an in situ perfusion experiment using rat jejunum plus ileum and questioned the nature of the transporter responsible for its entry into intestinal cells. The discrepancy may be a result of the difference in employed models (Caco-2 cells vs. rat intestine) and/or test flavonoid compounds. Thus, future studies are warranted to examine whether our data can be replicated in different modeled cell lines and with flavonoids with diverse glycoside moieties.

The inhibitory potency of phloridzin and phloretin at the same concentration on quercitrin transport appeared slightly different even though both had a complete inhibition in the early stage of quercitrin transport from the apical to the basolateral membrane. Zou et al. (30) reported that both SGLT1 and GLUT2 were involved in the transport of cyanidin-3-glucoside. Consistent with this finding, our results suggest that both of them are involved in quercitrin transport in the same cell model. However, it should be noted that our results did not provide adequate information for the speculation of transporters’ location and mechanism of actions. Especially, the expression of GLUT2 in the apical membrane of Caco2 that was reported in studies when the cells are exposed to a high (>25 mmol/L) glucose condition, must be determined in future studies. We speculate from the waning potency along with the incubation time that the inhibitory regulation of phloridzin and phloretin on the glucose transporters may be reversible, probably via a competitive mechanism for binding to the same structure of transporters between the inhibitors and quercitrin (39). Alternatively, the numbers of transporters may be increased by the presence of quercitrin in the apical and basolateral membranes, similar to the mechanism by which glucose increases the incorporation of GLUT2 transporters in the apical membrane of the enterocytes, particularly after a meal with abundant digestible carbohydrates (40). Such speculation requires further studies to examine the expression of transporters in protein and mRNA levels. Additionally, experiments using a sodium-free buffer with and without phloretin can be conducted to measure the extent of GLUT2s’ contribution to the transport of quercitrin and other flavonoid glycosides across the apical membrane. Furthermore, given that only one dose (0.1 mmol/L) of phloridzin and phloretin and one pretreatment duration were employed in this study, future studies are warranted to elucidate their impact on quercitrin transport across Caco2 cells with a consideration of dose and pretreatment duration.

The biological effects of flavonoids in target tissues are mainly exerted by their metabolites derived from phase II metabolism, primarily glucuronidation, sulfation, and methylation (3, 7). Because authenticated standards for quercetin glucuronides and sulfates are not readily available, we only measured quercetin and methylated quercetin, isorhamnetin and tamarixetin, in the collected samples. The detection of quercetin inside of the cells in the uptake experiment support the hydrolysis of quercitrin in the removal of the rhamnose moiety from quercetin, probably mediated by the broad-specific enterocyte β-glucosidase and/or the lactase phloridzin hydrolase (38, 41, 42). Furthermore, the observation that quercetin was only found inside the cells in the uptake experiment but not in the receiving chambers in the transport experiments suggests that quercetin derived from the hydrolysis of quercitrin is efficiently transformed by the phase II enzymes and then transported to the opposite side of the administration chamber (43, 44). The decrease in both methylated quercetin metabolites noted after 60 min of the uptake experiment implicates the fact that the methylated products may serve as the substrate for other phase II enzymes, UDP-glucuronosyl transferase and sulfotransferase (45). Further studies are warranted to substantiate this speculation.

## Conclusions

Flavonoids are generally present in nature with glycoside conjugate(s) in their hydroxyl moieties. While it is commonly appreciated that the removal of glycoside conjugate(s) is a prerequisite for their absorption in the gastrointestinal tract, some studies reported flavonoid glycosides could be absorbed intact. In this *in vitro* study, we found that quercitrin, a quercetin glycoside, was transported intact across the Caco-2 cell monolayer, but was also subjected to metabolism, including deglycosylation and methylation, in the modeled enterocytes. Our results also confirmed that both SGLT1 and GLUT2 are involved in the transport of quercitrin even though the exact mechanism by which SGLT1 and GLUT2 mediate the transport remains to be elucidated. Therefore, a study is warranted to investigate whether GLUT2 is expressed in the apical membrane and involved in quercitrin’s transport. The early complete inhibition of quercitrin transport when quercitrin was co-incubated with the inhibitors of glucose transporters, that is phloridzin and phloretin, suggests a competitive inhibitory mechanism. In conclusion, our results demonstrate that quercitrin, a flavonoid glycoside, can be absorbed intact and then effluxed out of enterocytes through both apical and basolateral membranes probably via SGLT1 and GLUT2. Future studies are warranted to examine whether the same phenomenon can occur in other flavonoid glycosides and can be replicated in different cell lines.
